# Rigid Minds, Fragile Meals: a literature review of shared mechanisms, challenges, and management approaches to comorbid autism and eating disorders

**DOI:** 10.1192/j.eurpsy.2026.12024

**Published:** 2026-07-31

**Authors:** S. Velhote, F. S. Nogueira, G. R. Santos, B. Ribeiro, C. Rio

**Affiliations:** 1 CRI - Saúde Mental; 2 DPSM, Unidade Local de Saúde Tâmega e Sousa, Penafiel, Portugal

## Abstract

**Introduction:**

Growing evidence shows a significant link between ASD/autistic traits and FEDs, which present unique challenges when comorbid. Autism Spectrum Disorder (ASD) is a neurodevelopmental disorder characterized by persistent difficulties with social skills, communication and restricted, repetitive patterns of thinking and behaviour, while autistic traits represent manifestations of these features that don’t meet criteria for an ASD diagnosis. Feeding and eating disorders (FEDs) are a group of disorders where abnormal eating behaviours impair the individual’s health and functioning, and they include Avoidant/Restrictive Intake Disorder, anorexia nervosa, bulimia nervosa, and binge eating disorder.

**Objectives:**

This paper aims to review current understanding of the association between ASD/autistic traits and FEDs, its underlying mechanisms, and guidelines for management and evidence-based treatment of individuals with comorbid ASD and FEDs.

**Methods:**

For this literature review, several databases and scientific journals (PubMed, Google Scholar, World Psychiatry, The Lancet Psychiatry, Jama Psychiatry, European Psychiatry, American Journal of Psychiatry, The British Journal of Psychiatry) were searched using the keywords autism spectrum disorder, autism traits, eating disorders, anorexia nervosa. Articles that matched the aims for this paper were selected and reviewed.

**Results:**

Autistic traits appear to be overrepresented in individuals FEDs, particularly ARFID, but also classic eating disorders such as anorexia nervosa. Some explanations surround shared neurobiological mechanisms such as genetic overlap, altered sensory processing, and cognitive rigidity, with emerging evidence implicating gut-brain and immune alterations. Moreover, psychosocial contributors further modify risk and clinical presentation, and include family mealtime dynamics, parental feeding practices, and the impact of comorbid psychiatric disorders.

Management of FEDs in individuals with ASD/autistic traits is uniquely challenging. Individuals with comorbid ASD and FEDs tend to have poorer treatment adherence and outcomes. Proposed guidelines for management include screening assessment of autistic traits in individuals with FEDs, and multidisciplinary, individualized approaches tailored for neurodiversity, such as adaptations for sensory sensitivities, cognitive rigidity, and differences in social-communication.

**Conclusions:**

Increasing evidence supports a significant association between ASD/autistic traits and FEDs and propose underlying mechanisms with neurobiological and psychosocial basis. This population is uniquely challenging and effective approaches are still uncharted territory. There is a striking need for further research to optimize assessment tools, develop tailored interventions, and improve outcomes in this complex population.

**Disclosure of Interest:**

None Declared

